# Effects of health-promoting school strategy on dental plaque control and preventive behaviors in schoolchildren in high-caries, rural areas of Taiwan: a quasi-experimental design

**DOI:** 10.1186/s12903-021-01927-z

**Published:** 2021-11-08

**Authors:** Chun-Ting Wei, Kai-Yang Lo, Yi-Ching Lin, Chih-Yang Hu, Fu-Li Chen, Hsiao-Ling Huang

**Affiliations:** 1 Department of Oral Hygiene, College of Dental Medicine, Kaohsiung Medical University, 100 Shih-Chuan 1st Road, Sanmin Dist., Kaohsiung City, 80708 Taiwan; 2 Department of Oral Hygiene, Tzu Hui Institute of Technology, No.367 Sanmin Rd., Nanzhou, Pingtung 92641 Taiwan; 3 Center for Physical and Health Education, National Sun Yat-Sen University, No. 70 Lien-hai Rd., Kaohsiung, 80424 Taiwan; 4 School of Dentistry, College of Dental Medicine, Kaohsiung Medical University, 100 Shih-Chuan 1st Road, Sanmin Dist., Kaohsiung City, 80708 Taiwan; 5 School of Public Health, Health Sciences Center, Louisiana State University, 2020 Gravier Street, New Orleans, LA 70112 USA; 6 Department of Public Health, Fu Jen Catholic University, No. 510, Zhongzheng Rd., Xinzhuang Dist., New Taipei City, 242062 Taiwan

**Keywords:** Dental health, Health-promoting school, Preventive behavior

## Abstract

**Background:**

We evaluated the effects of health-promoting school (HPS) strategy on plaque control and behavior change in high-caries schoolchildren in Taitung, Taiwan.

**Methods:**

A quasi-experimental design was adopted; six intervention schools (intervention group [IG]) and six comparison schools (comparison group [CG]) were selected from elementary schools with higher-than-average caries rates (> 68%). The IG was selected using cluster sampling, and the CG was selected to match the IG. In total, the IG and CG groups included 166 and 174 children each. The selected schools implemented the HPS framework for 3 months in the 2019 academic year. An oral examination of dental plaque was administered, and a self-administered questionnaire regarding knowledge, attitude, self-efficacy, and behaviors was distributed at baseline and at 2-week follow-up. A linear and logistic regression model using generalized estimating equations (GEEs) was used to analyze the differences between baseline and the follow-up data.

**Results:**

Compared with the CG, the IG had a greater reduction in plaque index among second graders (β =  − 0.36) and plaque control record scores among second, fourth, and sixth graders (β =  − 27.48, − 26.04, and − 18.38, respectively). The IG also exhibited a greater increase at follow-up with respect to oral health–related knowledge among second graders and fourth graders (β = 1.46 and β = 0.92, respectively), attitude toward oral hygiene behaviors among sixth graders (β = 1.78), and self-efficacy regarding flossing for sixth graders (β = 1.43). Sixth graders in the IG were significantly more likely to brush before sleeping (adjusted odds ratio [aOR] = 2.99) and use fluoride toothpaste (aOR = 5.88) than those in the CG.

**Conclusions:**

The HPS strategy was effective in reducing dental plaque and promoting preventing behaviors in rural high-caries schoolchildren.

**Supplementary Information:**

The online version contains supplementary material available at 10.1186/s12903-021-01927-z.

## Introduction

Dental caries in children is a major public health concern in Taiwan [1]. Children affected by caries can experience pain and sleeping disorders, affecting school attendance and performance, eating habits, body weight, and growth [2, 3]. In 2012, the decayed, missing, and filled (DMF) teeth index for 12-year-old children in Taiwan was 2.5; this is higher than the global average for DMF as reported in a World Health Organization survey [4]. Studies in the United States and Australia have noted the effects of geographic location on caries prevalence [5, 6]. Urban–rural disparities in health care resources are prominent in Taiwan, especially in dental care, which is mostly concentrated in urban areas. Taitung County is located primarily on the island’s southeastern coast, which is somewhat rural and has the highest percentage of Taiwan’s aboriginal population (35.5%). DMF scores in 12-year-old children in eastern Taiwan are 1.52 times higher than those in the western counties [7]. In 2017, the prevalence of dental caries among schoolchildren in Taiwan was 61.1%. The prevalence in Taitung was 68.8% [8]. Compared with urban areas, schoolchildren in remote areas seriously lack oral health care provision, and access to medical resources and information are also inferior to those in urban areas. Eight townships in Taitung County had no dental clinic, accounting for 14.5% of Taiwan’s townships without dental clinics [9]. The generally low socioeconomic status and educational level of the aborigines also may have led to lower oral health literacy and poor oral health behaviors, causing more serious dental caries [10]. To prevent dental caries in schoolchildren, Taiwan’s Ministry of Health and Welfare committed to promoting oral health, including tooth brushing after meals, application of fluoride mouthwash in school, and free fissure sealing. Free preventive fluoride vanish application twice a year for children under 6 years old, with intervals no shorter than 180 days. In addition, free vanish was provided for schoolchildren aged under 12 in remote areas [11].

Dental caries forms through an interaction over time between acid-producing bacteria, carbohydrate, and many host factors, which create oral environment in low pH, resulting in mineral loss from the teeth [12, 13]. A major cause of dental caries is dental plaque, which often results from poor oral health–related behaviors, dietary habits, and a lack of appropriate oral health knowledge among children [14, 15]. Parents’ low level of oral health knowledge, attitude toward oral health, and socioeconomic status were risk factors for dental caries in children [16, 17]. Furthermore, regular dental visits and limits on the consumption of cariogenic products imposed by parents were associated with children’s caries status [18, 19]. Fluoride is useful in caries prevention. It is effective in promoting enamel caries lesion remineralisation and fluoridation and inhibiting bacterial activity in dental plaque [20, 21]. A longitudinal study in South Korea demonstrated that increased fluoride toothpaste and dental sealant use contributed to a decline in dental caries [22].

Because children spend the majority of their time there, school is an ideal setting for health promotion [23]. A health-promoting school (HPS) is a school that aims to strengthen a school’s capacity as a healthy living, learning, and working environment [24]. The six domains of the HPS framework were school policies, physical environment, social environment, health-related skills, community relationships, and health services [25, 26]. This is demonstrated by the effectiveness of HPS programs in other health-related categories, such as diet, weight, and vision [27, 28]. It is vital to improve children’s oral health through health promotion at an early stage, because such promotion received at primary school-age may affect health-related behavior later in life [29]. A school-based supportive environment was significantly associated with children’s oral health status [30]. Previous researches have mostly come in the form of curricular teaching intervention or a single domain related to oral health promotion [31, 32]. Only one study using HPS involved a 3-year oral health promotion program intended to reduce children’s new caries incidence, improve oral hygiene, and establish the habit of oral health behaviors [29]. The present study is the only study that executed intervention based on six domains of HPS and involved all elementary grade levels. We aimed to evaluate the effectiveness of HPS strategy intervention for dental plaque control and for the promotion of caries preventive behaviors in schools with a high prevalence of caries among students living in rural areas.

## Methods

### Design

All methods were carried out in accordance with the CONSORT guidelines and regulations [33]. A quasi-experimental design was employed. Cluster sampling was used, where the sampling unit is the school for this study. Six intervention schools were selected based on a high prevalence rate of caries, defined as schools with a dental caries rate higher than 68% (higher than the prevalence of dental caries in Taitung). To form the comparison group (CG), students with similar demographic backgrounds from six schools (located in the same geographic area) with similar caries prevalence were selected. All students attending the selected schools were invited to participate in the study.

### Participants

**Fig. 1:**
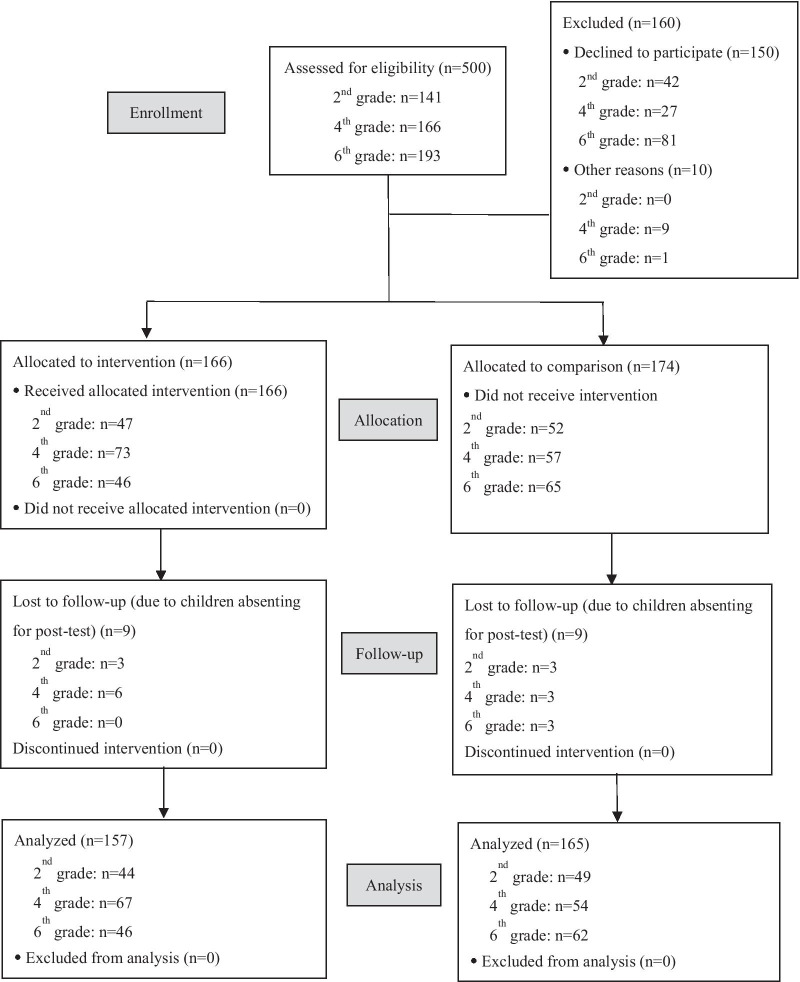
Flowchart illustrating how the analysis was conducted

Students with disabilities and students who declined to participate in the program were excluded. Sample size calculation was based on the plaque index (PI) [effect size (ES) = 0.5, *p* < 0.05, power = 0.8, 20% dropout]. Students who were absent in the posttest were considered to be lost to follow-up. In total, 166 and 174 children aged 7–12 from the IG and the CG (overall response rate 68%) were recruited, respectively (Fig. 1).

### Instruments

Dental plaque information was collected through oral examination. Oral examinations were conducted by two calibrated dentists. They received training before the oral examination to ensure that the examinations were consistent. The kappa coefficient for inter-rater reliability was 0.91. Oral examinations were performed using mirrors, a probe, and a flashlight. Plaque control record (PCR) scores were recorded by a dental hygienist. The questionnaires (Additional file 1: Tables S1–S3) regarding oral health knowledge, attitude, self-efficacy, and behavior was developed and revised based on the survey of the K-12 Education Administration, an agency within Taiwan’s Ministry of Education [34]. To ensure that the content was understood by our respondents, the questionnaire was piloted among a convenience sample of second, fourth, and sixth graders in Taitung. To enhance clarity and age appropriateness, items were revised as required according to the results of the pilot test. Furthermore, five experts reviewed the questionnaires to assess their fit with our topic of investigation, the clarity of the title, and the coverage of the content. The content validity index values for the second, fourth, and sixth grade questionnaires were 1.00, 0.995 and 0.997, respectively.

### Outcome

#### Plaque index

Recordings were made according to World Health Organization methods [35]. Six index teeth [16, 12(52), 24(64), 44(84), 32(72), and 36] were evaluated and assigned a score of 0 to 3 based on the following criteria: 0 = no plaque; 1 = a film of plaque adhering to the free gingival margin and adjacent area of the tooth; 2 = moderate accumulation of soft deposits within the gingival pocket or on the tooth and gingival margin that can be seen with the naked eye; 3 = abundance of soft matter within the gingival pocket or on the tooth and gingival margin. The PI was calculated by summing the average score of each tooth and dividing it by the number of teeth [35].

#### Plaque control record

The children’s teeth were coated using a disclosing agent liquid, and plaque adherence to teeth was recorded. PCR scores ranged from 0 to 100%. Each tooth was divided into six surfaces and the PCR scores were calculated based on the proportion of the tooth surface that was covered in plaque [36].

#### Oral health-related knowledge

Nine statements were used to determine the oral health-related knowledge of second graders (e.g., “Drinking sweet drinks will cause tooth decay”); scores ranged from 0 to 9. Eight statements were used to measure the oral health–related knowledge of fourth graders (e.g., “Fluoride can prevent tooth decay”); scores ranged from 0 to 8. Nine statements were used to measure related knowledge in sixth graders (e.g., “Dental floss is the best tool for cleaning between the teeth”); scores ranged from 0 to 9. Possible responses included “True,” “False,” and “I don’t know”; a high score indicated a high degree of knowledge. The Kuder-Richardson 20 (KR-20) coefficients were 0.59, 0.59, and 0.63 for second, fourth, and sixth graders, respectively.

#### Attitude toward oral hygiene

Nine statements were used to measure the attitudes of sixth graders, including three positive items (e.g., “I think brushing my teeth right after a meal is important”) and six negative items (e.g., “I think brushing my teeth is very troublesome”). Positive items were scored on a 4-point scale ranging from 1 (strongly disagree) to 4 (strongly agree) and negative items were scored in the opposite way. Total scores ranged from 4 to 36: a high score indicated a positive attitude toward oral hygiene. Cronbach’s α was 0.68 for this scale.

#### Self-efficacy toward oral hygiene

Four statements were used to measure self-efficacy toward toothbrushing in second, fourth, and sixth graders (e.g., “I am confident that I will brush my teeth before going to bed every night”). Each item was scored on a 4-point scale ranging from 1 (*strongly disagree*) to 4 (*strongly agree*). Scores ranged from 4 to 36: a high score indicated a high degree of self-efficacy. The Cronbach’s α was 0.72, 0.86, and 0.82 for second, fourth and sixth graders, respectively.

Four statements were used to measure self-efficacy toward flossing in sixth graders (e.g., “I am confident that I will use dental floss once a day”). Each item was scored on a 4-point scale ranging from 1 (*strongly disagree*) to 4 (*strongly agree*). Scores ranged from 4 to 36: a high score indicated a high degree of self-efficacy. The Cronbach’s α was 0.86 for this scale.

#### Oral health behavior

The question “I brush my teeth before going to bed” was used to assess toothbrushing behavior. Possible responses were “always,” “sometimes,” and “never.” Dentist visits were assessed among fourth and sixth graders using the question “How long has it been since my last visit to the dentist?” Possible responses were “I have never been,” “more than 6 months,” and “less than 6 months.” Flossing behavior was assessed among sixth graders using the question “How many times have I used floss to clean my teeth in the last 7 days?” Possible responses were “None,” “1 to 3 times,” “4 to 6 times,” “once a day,” “more than once a day,” and “not sure.”

### Intervention

HPS intervention strategies (see Additional file 2: Table S4) were implemented for 3 months during a semester in 2019. In the IG, one trained dental hygienist served as school liaison and visited the schools four times at 2-week intervals. Three dental hygienist received training before visiting the schools. The training content included the importance of preventive care and of consistency in curriculum content. The dental hygienists then formulated a suitable oral health promotion program. The following sections outline the deliberation on the six domains of the HPS framework:

#### School policies

The research team and each dental hygienist held a symposium with the school principal, administrators, school dentists, and school nurses, assessing the schoolchildren’s caries statuses and formulating suitable health policies accordingly. School policies included brushing teeth for 3 min while sitting in the classroom after lunch and restricting sugary snack and drink consumption between meals on school property. The duty of school teachers was to supervise children brushing their teeth after lunch. The dental hygienists not only provided support for the functioning of the school health policy committee but also directed more attention to issues related to children’s oral health.

#### Physical environment

The physical environment included the campus environment and all facilities and appliances. The dental hygienist monitored the suitability of the schoolchildren’s toothbrushing tools, including small-head and soft-bristle toothbrushes, and ensured that the level of fluoride reached more than 1000 ppm when brushing and that 0.2% fluoride mouthwash was used once a week. All oral hygiene supplies, including toothbrushes, dentifrices and fluoride mouthwash, were standardized for all schools [37]. Each dental hygienist also checked whether all schools had proper oral hygiene supplies, teaching aids, and tooth models and an adequate number of hand-washing stations. The dental hygienists provided schools with free fluoride toothpaste, media tools, such as toothbrushing videos and posters for oral health promotion.

#### Social environment

The purpose of the social environment is to establish a supportive system to train seed teachers and student peer leaders to become role models. The school teachers’ duties included supervising the correctness of children’s toothbrushing after meals, checking the suitability of toothbrushing tools, and reminding parents to require children to both brush their teeth before going to bed and change their cleaning tools regularly. Furthermore, schools established a reward system to encourage students to maintain good oral health behaviors and to establish students’ oral hygiene habits.

#### Health-related skills

To improve oral health–related knowledge and skills, we held one 40-min classroom teaching session for students regarding oral health education, one oral health education lecture for parents, and one dental hygienist-led oral health empowerment workshop for teachers. Standardized lecture slides were prepared based on the curriculum. The topics for classroom teaching included tooth function, factors related to tooth decay, healthy diet choices, choice of toothbrush and toothpaste, knowledge of fluoride, regular dental visits, and toothbrushing skills. The lecture topics for teachers and parents included the importance of children’s oral health, risk factors for dental caries, and methods for preventing dental caries. The content of all the course emphasized the application and importance of fluoride. Through carefully-designed teaching activities, all participants (i.e., students, parents or guardians, and teachers) improved their oral health awareness and strengthened their oral health skills.

#### Health services

In terms of health services, school nurses were advised to monitor the children’s caries status and provide individualized caries counseling for high-risk children. Free school-based fluoride varnish application and fissure sealing were also provided. In addition, schools were expected to provide sustainable funding for oral health instruments and to purchase oral health appliances, thereby providing schoolchildren with improved health services.

#### Community relationships

Schools connected with community and health care services using community resources to establish a supportive environment. Schools communicated with stores in their local communities and set up sugar-free stores to reduce access to sugary food and drinks. In addition, schools cooperated with local dental clinics. These dental clinics reduced medical costs for local schools and assigned dentists to schools to provide medical services for schoolchildren.

### Data collection

The questionnaire with identical questions regarding children’s level of knowledge, attitude, self-efficacy, and oral health behavior was administered at baseline and 2-week follow-up. However, first grader may not understand all items/questions in the questionnaire due to low literacy level. Therefore, we choose second, fourth, and sixth graders to fill the questionnaires and oral examination. Members of the research team administered pre- and post-test questionnaires to students in classrooms during the spring semester of the 2019 academic year. The PI levels were collected by dentists and the PCR scores were collected by dental hygienists.

### Data analysis

We used Fisher’s exact test and the Mann–Whitney U test to analyze the baseline comparison between the IG and CG. PI, PCR score, knowledge, self-efficacy, and attitude scores within groups were measured using paired *t* tests, and the differences between groups were measured using independent *t* tests. Linear regression with generalized estimating equations (GEEs) was used to calculate the regression coefficient between groups. The ES of continuous variables was calculated using Cohen’s *d* [38] as the mean difference between pretests and posttests. An effect of 0.20 is small, one of 0.50 is moderate, and one of 0.80 is large. Logistic regression in GEE was used to assess the effects of the intervention on oral health behaviors. Statistical significance was set at *p* < 0.05. All data were analyzed using Stata version 13.1 (Stata Corp LP, College Station, Texas, USA).

## Results

Table 1 shows the baseline information for the two groups arranged by grade. No significant differences were observed in gender or PI and PCR scores in second, fourth and sixth grade (all *p* > 0.05).

**Table 1:** Baseline information for the intervention and comparison groups

	Second grade				Fourth grade				Sixth grade			
	IG		CG		IG		CG		IG		CG	
	n	%	n	%	n	%	n	%	n	%	n	%
*Gender*^a^												
Male	28	63.6	22	44.9	40	59.7	32	59.3	18	39.1	27	43.5
Female	16	36.4	27	55.1	27	40.3	22	40.7	28	60.9	35	56.5
PI(M ± SD)^b^	1.7 ± 0.5		1.5 ± 0.6		1.7 ± 0.5		1.5 ± 0.9		1.5 ± 0.6		1.3 ± 0.5	
PCR(M ± SD)^b^	46.6 ± 18.3		45.5 ± 22.3		43.2 ± 20.8		41.1 ± 21.0		43.4 ± 22.1		39.7 ± 18.2	

^a^Fisher’s exact test

^b^Mann-Whitney U test

PI = Plaque index

PCR = Plaque control record

Table 2 shows oral health knowledge, attitude, and self-efficacy for each group, and the regression coefficient between groups. Compared with the CG, the IG exhibited a greater increase at follow-up with respect to oral health–related knowledge for second graders and fourth graders (β = 1.46, 95% confidence interval [CI] = 0.76 to 2.15, ES = 0.85 and β = 0.92, 95% CI = 0.31 to 1.53, ES = 0.54, respectively), attitude toward oral hygiene behaviors for sixth graders (β = 1.78, 95% CI = 0.27 to 3.28; ES = 0.45), and self-efficacy regarding flossing for sixth graders (β = 1.43, 95% CI = 0.30 to 2.56; ES = 0.47) than the CG did.

**Table 2:** Regression-estimated mean difference of oral health knowledge, attitude, and self-efficacy among schoolchildren between the groups

Variables	IG			CG			Reg. coef.* (95% CI)	ES
	Pre-test	Post-test	Diff	Pre-test	Post-test	Diff		
	M ± SD	M ± SD	diff ± SD	M ± SD	M ± SD	diff ± SD		
*Second grade*								
Knowledge (0–9)	5.4 ± 1.7	6.3 ± 1.5	0.9 ± 1.6	5.7 ± 1.8	5.1 ± 1.9	− 0.6 ± 1.8	1.46 (0.76, 2.15)	0.85
Self-efficacy regarding toothbrushing (4–36)	14.5 ± 2.1	14.8 ± 1.7	0.3 ± 2.6	13.8 ± 2.7	14.0 ± 2.6	0.2 ± 3.2	0.09 (− 1.08, 1.29)	0.03
*Fourth grade*								
Knowledge (0–8)	5.3 ± 1.6	6.4 ± 1.3	1.1 ± 1.6	5.0 ± 1.9	5.2 ± 2.0	0.2 ± 1.9	0.92 (0.31, 1.53)	0.54
Self-efficacy regarding toothbrushing (4–36)	12.8 ± 2.9	12.5 ± 3.6	− 0.4 ± 4.2	12.3 ± 2.9	12.2 ± 3.6	− 0.1 ± 4.2	− 0.27 (− 1.76, 1.23)	− 0.06
*Sixth grade*								
Knowledge (0–9)	5.2 ± 2.4	6.4 ± 2.1	1.3 ± 2.0	5.0 ± 2.4	5.7 ± 2.2	0.7 ± 2.0	0.59 (− 0.14, 1.34)	0.28
Attitude (4–36)	28.0 ± 4.7	29.6 ± 4.6	1.6 ± 4.2	28.8 ± 3.8	28.7 ± 4.7	− 0.2 ± 3.8	1.78 (0.27, 3.28)	0.45
Self-efficacy regarding toothbrushing (4–36)	12.6 ± 2.3	13.5 ± 2.8	0.9 ± 3.1	13.5 ± 2.5	13.2 ± 3.1	− 0.2 ± 3.7	1.10 (− 0.22, 2.41)	0.31
Self-efficacy regarding flossing (4–36)	11.9 ± 2.9	13.2 ± 2.8	1.4 ± 2.8	12.1 ± 2.6	12.0 ± 3.4	− 0.1 ± 3.2	1.43 (0.30, 2.56)	0.47

^*^Reg. coef.: Adjusted for gender by linear regression with generalized estimating equations (GEE); Reference: Control group × Time (baseline)

Effect size based on mean difference between groups: Cohen’d, 0.20 is small, 0.50 is moderate, and 0.80 is large

Table 3 illustrates the difference in caries preventive behaviors among schoolchildren between the groups. For sixth graders, the percentage of participants in the IG who brushed before sleeping was 45.65% in pre-test and 67.39% in post-test. Relatively, the percentage of participants in the CG who brushed before sleeping was 67.74% in pre-test and 64.52% in post-test. Participants in the IG were more likely than those in the CG to brush before sleeping (adjusted odds ratio [aOR] = 2.99, 95% CI = 1.50 to 5.99) after intervention. In the IG, the percentage of participants who used fluoride toothpaste was 36.96% in pre-test and 78.26% in post-test. Relatively, the percentage of participants in the CG who used fluoride toothpaste was 35.48% in pre-test and 37.1% in post-test. After intervention, participants in the IG were more likely than those in the CG to use fluoride toothpaste (aOR = 5.88, 95% CI = 2.31 to 14.93).

**Table 3:** Regression-estimated difference in behavior among schoolchildren between the groups

Variables	IG		CG		aOR* (95% CI)
	n	%	n	%	
*Second grader*					
Brush before sleeping					1.00 (0.38–2.61)
Pre-test	30	68.18	35	71.43	
Post-test	31	70.45	36	73.47	
Toothbrush replaced regularly					1.70 (0.31–9.40)
Pre-test	36	81.82	32	65.31	
Post-test	42	95.45	41	83.67	
Brush with fluoride toothpaste					8.47 (0.90–79.75)
Pre-test	41	93.18	47	95.92	
Post-test	43	97.73	44	89.8	
*Fourth grader*					
Brush before sleeping					0.78 (0.34–1.77)
Pre-test	33	49.25	24	44.44	
Post-test	40	59.7	33	61.11	
Toothbrush replaced regularly					1.15 (0.25–5.34)
Pre-test	59	88.06	47	87.04	
Post-test	62	92.54	49	90.74	
Brush with fluoride toothpaste					1.82 (0.64–5.23)
Pre-test	29	43.28	23	42.59	
Post-test	49	73.13	32	59.26	
Brush more than 2 min					1.07 (0.41–2.80)
Pre-test	28	41.79	16	29.63	
Post-test	36	53.73	21	38.89	
Regular dental visits					1.74 (0.76–3.98)
Pre-test	28	41.79	28	51.85	
Post-test	42	62.69	32	59.26	
Sixth grader					
Brush before sleeping					2.99 (1.50–5.99)
Pre-test	21	45.65	42	67.74	
Post-test	31	67.39	40	64.52	
Toothbrush replaced regularly					0.63 (0.82–4.89)
Pre-test	41	89.13	60	96.77	
Post-test	42	91.3	61	98.39	
Brush with fluoride toothpaste					5.88 (2.31–14.93)
Pre-test	17	36.96	22	35.48	
Post-test	36	78.26	23	37.1	
Brush more than 2 min					1.15 (0.55–2.39)
Pre-test	21	45.65	16	25.81	
Post-test	27	58.7	21	33.87	
Regular dental visits					1.26 (0.49–3.23)
Pre-test	20	43.48	25	40.32	
Post-test	27	58.7	31	50	
Use floss at least once/day					2.70 (0.66–11.04)
Pre-test	7	15.22	6	9.68	
Post-test	15	32.61	6	9.68	

^*^aOR: Adjusted for gender by logistic regression in GEE; Reference: Control group × Time (baseline)

Table 4 presents the changes in dental PI levels between the groups of schoolchildren by grade. The IG had a greater reduction in the level of PI (β =  − 0.36, 95% CI =  − 0.59 to − 0.13; ES = 0.63) for second graders than the CG did. Moreover, the IG had a greater reduction in level of PCR for second graders, fourth graders, and sixth graders (β =  − 27.48, 95% CI =  − 37.16 to − 17.80; ES = 1.14; β =  − 26.04, 95% CI =  − 33.64 to − 18.44; ES = 1.22; β =  − 18.38, 95% CI =  − 26.57 to − 10.19; ES = 0.85, respectively).

**Table 4:** Regression-estimated mean differences of plaque index and plaque control record among schoolchildren by grade between the groups

Variables	IG			CG			Reg. coef.* (95% CI)	ES
	Pre-test	Post-test	Diff	Pre-test	Post-test	Diff		
	M ± SD	M ± SD	diff ± SD	M ± SD	M ± SD	diff ± SD		
*Second grade*								
PI	1.7 ± 0.5	1.1 ± 0.3	− 0.6 ± 0.5	1.5 ± 0.6	1.2 ± 0.5	− 0.3 ± 0.6	− 0.36 (− 0.59, − 0.13)	0.63
PCR	46.6 ± 18.3	42.2 ± 16.1	− 4.4 ± 23.0	45.5 ± 22.3	68.6 ± 18.1	23.1 ± 24.9	− 27.48 (− 37.16, − 17.80)	1.14
*Fourth grade*								
PI	1.7 ± 0.5	1.2 ± 0.5	− 0.5 ± 0.6	1.5 ± 0.6	1.1 ± 0.4	− 0.5 ± 0.6	− 0.04 (− 0.26, 0.18)	0.07
PCR	43.2 ± 20.8	45.3 ± 16.0	2.1 ± 23.3	41.1 ± 21.1	69.3 ± 18.1	28.2 ± 18.7	− 26.04 (− 33.64, − 18.44)	1.22
*Sixth grade*								
PI	1.5 ± 0.6	1.2 ± 0.5	− 0.3 ± 0.6	1.3 ± 0.5	1.1 ± 0.5	− 0.2 ± 0.5	− 0.11 (− 0.31, 0.09)	0.21
PCR	43.4 ± 22.1	49.1 ± 17.8	5.7 ± 23.5	39.7 ± 18.2	63.7 ± 17.0	24.0 ± 20.2	− 18.38 (− 26.57, − 10.19)	0.85

^*^Reg. coef.: Adjusted for gender by linear regression in GEE; Reference: Control group × Time (baseline)

Effect size based on mean difference between groups: Cohen’d, 0.20 is small, 0.50 is moderate, and 0.80 is large

## Discussion

The HPS framework for intervention improved schoolchildren’s plaque control and oral health–related knowledge, and the framework improved sixth graders’ self-efficacy with regard to flossing behavior and caries preventive behaviors. Decreases in PCR scores were more significant among second, fourth, and sixth graders in the IG than among those in the CG. The PI score in the IG decreased significantly among second graders after intervention. With regard to preventive behavior against caries, the sixth graders in the IG were more likely to brush before sleeping and brush with fluoride toothpaste than those in the CG.

Our findings are consistent with previous studies [29, 30], which showed that school-based programs could improve oral hygiene and establish positive oral health behaviors among schoolchildren. Studies have indicated that oral health education by dental professionals is an effective method of reducing dental PI and improving oral health knowledge among children [31, 32]. School teachers are also suitable individuals for delivering oral health education to schoolchildren. Trained teachers have a positive effect on schoolchildren’s oral health [39]. Moreover, one study demonstrated that short-term health education in schools could significantly reduce PCR scores among schoolchildren [40]. This study involved not only delivering oral health education to schoolchildren but also training teachers, endowing them with professional knowledge. Teachers were vital agents in the program and helped children to improve their oral hygiene behaviors.

The results showed that the difference in PI between the IG and CG was smaller among fourth and sixth graders than among second graders. It is reasonable to assume that fourth and sixth graders had more effective brushing skills than second graders because of their improved motor development, leading to their lower PI scores at baseline; thus, the difference in PI would be less among fourth and sixth graders than among second graders. Furthermore, our findings indicated that students in higher grades were more likely to engage in oral health behaviors and that they made more progress in self-efficacy than those in lower and middle grades. This finding was similar to those of previous studies that showed that children with high self-efficacy are more likely to engage in this behavior [41, 42]. In addition, as students grow older, they are better able to understand the relationship between health concepts and their behaviors; consequently, they change their behaviors to promote health [43].

We found that behaviors at home, including brushing using fluoride toothpaste and brushing teeth before going to bed, had improved. Oral health education targeting both the school and home environments could improve children’s toothbrushing behavior [44]. In the present study, classroom teaching session for students and empowerment workshop for teachers emphasized the importance of fluoride application and toothbrushing before going to bed. Furthermore, a parental education session was held to instruct parents about the importance of fluoride and brushing before going to bed, which may have increased their monitoring of brushing teeth before going to bed and supervising the oral health habits of their children. One study suggested that children’s oral health education should include parents’ roles in improving their children’s oral health, because parents' attitudes toward oral health were associated with children's oral health behaviors [45].

In our study, the level of knowledge among second, fourth, and sixth graders in the IG increased more than that in the CG. Additionally, sixth grade children’s attitude toward oral hygiene had greater improvements in the IG than in the CG. Studies have shown that oral health education can improve schoolchildren’s oral health-related knowledge and attitudes [32, 46]. In one study, children’s toothbrushing behavior, oral health-related knowledge, and attitudes toward oral health improved simultaneously, suggesting that changes in knowledge, attitudes, and behavior were related [44]. Therefore, using intervention strategies to improve children’s knowledge and attitude should also have some effect on children’s oral health behaviors. Furthermore, our findings indicated that the HPS program improved self-efficacy with regard to flossing among sixth grade students in the IG. For sixth graders, the ES’s of the IG for self-efficacy with regard to toothbrushing and flossing were higher than those of the CG. However, second and fourth grade students had no changes in self-efficacy with regard to toothbrushing. Another study [44] demonstrated that oral health education programs could improve self-efficacy with regard to toothbrushing and flossing among children in upper grades. Lower-grade students may encounter difficulties in practicing new behaviors after learning new knowledge and skills, whereas upper-grade students with more developed minds and learning abilities showed greater confidence.

### Limitations

First, this study did not use a true experimental design, which may have led to selection bias. Second, surveys relied on self-reports; thus, these data were subject to report bias. However, some studies have shown that self-reported data are valid and reliable when the individuals’ privacy is protected [47, 48]. Finally, these findings should be generalized to other settings or populations with caution.

## Conclusions

The HPS strategy was effective in reducing plaque, improving oral health knowledge and self-efficacy with regard to dental flossing, and promoting caries-preventing behaviors among rural schoolchildren with a high-caries prevalence.

## Supplementary Information

**Additional file 1**. Items of knowledge, attitude, self-efficacy and behavior

**Additional file 2**. Intervention strategies based on health-promoting school model

## Data Availability

The datasets generated and/or analyzed during the current study are not publicly available due to maintain participant privacy and confidentiality requirements but are available from the corresponding author on reasonable request.

## Abbreviations

CG: Comparison Group
DMF: Decayed, missing, and filled
ES: Effect size
HPS: Health Promoting School
IG: Intervention Group
PCR: Plaque control record
PI: Plaque Index
