# Supplementary material for: Effects of health-promoting school strategy on dental plaque control and preventive behaviors in schoolchildren in high-caries, rural areas of Taiwan: a quasi-experimental design
Source: BMC Oral Health. 2021 Nov 8;21:573. doi: 10.1186/s12903-021-01927-z (PMC8573891; doi:10.1186/s12903-021-01927-z)
Supplement: Supplementary file 1 — Additional file 1. Items of knowledge, attitude, self-efficacy and behavior [file 12903_2021_1927_MOESM1_ESM.docx]

| Supplementary table S1. Items of knowledge, self-efficacy and behavior for second-grade students |
| --- |
| Items Questions |
| Knowledge  1. Drinking sweet drinks will cause tooth decay.  2. Incisors are rectangular in shape and used to cut food.  3. All of my teeth only grow back once.  4. Small head toothbrushes should be used when brushing my teeth.  5. Toothbrushes should be changed every 6 months.  6. Fluoride can prevent tooth decay.  7. Only visit a dentist if you have a toothache.  8. You should brush your teeth for more than 3 minutes.  9. Please identify the food that are bad for your teeth: soft drinks, milk tea, black tea, vegetables, cakes, apples, and pudding. |
| Self-efficacy regarding toothbrushing  1. Brushing and cleaning my teeth is _____ for me.  2. Brushing my teeth after lunch is _____ for me.  3. Brushing my teeth before going to bed is _____ for me.  4. Brushing my teeth after eating snacks is _____ for me. |
| Oral health behavior  1. I brush my teeth before going to bed.  2. Do I brush my teeth after lunch at school?  3. How often do I change my toothbrush every semester?  4. Do I use toothpaste when brushing my teeth at school?  5. Do I brush every tooth thoroughly when brushing my teeth?  6. How often do I drink sweet drinks such as Yakult, soft drinks, milk tea, and black tea every week at school?  7. How often do I eat snacks such as candies, biscuits, and chocolate every week at school?  8. Do I brush my teeth after eating sweet food? |
|  |

| Supplementary table S2. Items of knowledge, self-efficacy and behavior for fourth-grade students |
| --- |
| Items Questions |
| Knowledge  1. Small head toothbrushes should be used when brushing my teeth.  2. Incisors are rectangular in shape and used to cut food.  3. Only visit a dentist if you have a toothache.  4. All of my teeth only grow back once; the new teeth are called permanent teeth.  5. Fluoride can prevent tooth decay.  6. A dental checkup is required every 6 months.  7. You should brush your teeth for more than 3 minutes.  8. Brushing with a toothbrush with harder bristles can ensure cleaner teeth. |
| Self-efficacy regarding toothbrushing  1. I am confident that I can brush my teeth cleanly.  2. I am confident that I will brush my teeth after lunch.  3. I am confident that I will brush my teeth before going to bed every night.  4. I am confident that I will brush my teeth after I have eaten snacks. |
| Oral health behavior  1. I brush my teeth before going to bed.  2. Do I brush my teeth after lunch?  3. Do I use fluoride toothpaste (toothpaste with a fluoride concentration over 1000 parts per million) to brush my teeth?  4. On average, how much time do I spend to brush my teeth?  5. How often do I change my toothbrush every semester?  6. How often do I drink sweet drinks such as Yakult, soft drinks, milk tea, and black tea every week at school?  7. How often do I eat snacks such as candies, biscuits, and chocolate every week at school?  8. Do I ask my caregiver to take me to the dentist myself?  9. How long has it been since my last visit to the dentist? |
|  |

| Supplementary table S3. Items of knowledge, attitude, self-efficacy and behavior for sixth-grade students |
| --- |
| Items Questions |
| Knowledge  1. Only visit the dentist when you have a toothache; regular checkups are not necessary.  2. Tooth decay forms because of four factors: tooth, food, bacteria, and time.  3. A small head toothbrush should be used when brushing my teeth.  4. To prevent tooth decay, fluoride toothpaste must have a fluoride concentration over 1000 parts per million.  5. After using fluoride mouthwash, you should wait for at least 3 minutes before eating.  6. Inflamed gums are usually caused by irritation.  7. Dental floss is the best tool for cleaning between the teeth.  8. In the Bass method of brushing, the bristles are angled at 90 degrees to the surface of the teeth.  9. Brushing with a toothbrush with harder bristles can ensure cleaner teeth. |
| Attitude  1. I think that other illnesses, such as a cold or stomachache, are more serious than dental illnesses.  2. I think brushing my teeth is very troublesome.  3. I do not use dental floss because I think cleaning with dental floss is not pleasing to the eye.  4. I think it is fine to have tooth decay.  5. I do not have to use dental floss if I brush my teeth thoroughly every day.  6. I think using dental floss is very troublesome.  7. I avoid sweet food to protect the health of my teeth.  8. I think brushing my teeth right after a meal is important.  9. I think cleaning my teeth is as important as taking a shower every day. |
| Self-efficacy regarding toothbrushing  1. I am confident that I can brush my teeth cleanly.  2. I am confident that I will brush my teeth after lunch.  3. I am confident that I will brush my teeth before going to bed every night.  4. I am confident that I will brush my teeth after I have eaten snacks. |
| Self-efficacy regarding flossing  1. I am confident that I will use dental floss to clean my teeth.  2. I think using dental floss is easy.  3. I am confident that I will use dental floss once a day.  4. I am confident that I will use dental floss before I go to bed.  Oral health behavior  1. I brush my teeth before going to bed.  2. Do I brush my teeth after lunch at school?  3. Do I use fluoride toothpaste (toothpaste with a fluoride concentration over 1000 parts per million) to brush my teeth?  4. On average, how much time do I spend brushing my teeth?  5. How often do I change my toothbrush every semester?  6. How often do I drink sweet drinks such as Yakult, soft drinks, milk tea, and black tea every week at school?  7. How often do I eat snacks such as candies, biscuits, and chocolate every week at school?  8. How many times have I used floss to clean my teeth in the last 7 days?  9. Do I ask my caregiver to take me to the dentist myself?  10. How long has it been since my last visit to the dentist? |
|  |
