# Supplementary material for: Effects of health-promoting school strategy on dental plaque control and preventive behaviors in schoolchildren in high-caries, rural areas of Taiwan: a quasi-experimental design
Source: BMC Oral Health. 2021 Nov 8;21:573. doi: 10.1186/s12903-021-01927-z (PMC8573891; doi:10.1186/s12903-021-01927-z)
Supplement: Supplementary file 2 — Additional file 2. Intervention strategies based on health-promoting school model [file 12903_2021_1927_MOESM2_ESM.docx]

| Supplementary table S4. Intervention strategies based on health-promoting school model | |
| --- | --- |
| Health-promoting domain | Counselling-based intervention strategy |
| School policies | - Establish policies demanding that students brush their teeth after meals - Have students brush their teeth in their seats and use fluoride toothpaste - Improve the functioning of the school’s oral health committee |
| Physical environment | - Ensure the appropriateness of dental cleaning tools - Provide fluoride toothpaste - Purchase oral health consumables, teaching aids, and tooth models - Develop tooth brushing media, posters, and videos |
| Social environment | - Train teachers or tooth brushing helpers (students who remind their peers to brush) - Teachers supervising and reminding students to brush their teeth after meals - Parents reminding their children to brush their teeth and change their dental cleaning tools before going to bed |
| Health-related skills | - Educate students in class - Establish professional development programs for teachers - Host oral health lectures for parents and children |
| Health services | - Continuously track students’ tooth decay situations - Provide oral health products such as fluoride varnish and pit-and-fissure sealants - School health center designating funds for oral health |
| Community relationships | - Establish excellent sugar-free businesses in communities - Connect schools and health departments together |
